# Surface Dielectric Barrier Discharge plasma: a suitable measure against fungal plant pathogens

**DOI:** 10.1038/s41598-020-60461-0

**Published:** 2020-02-28

**Authors:** Paolo F. Ambrico, Milan Šimek, Caterina Rotolo, Massimo Morano, Angelantonio Minafra, Marianna Ambrico, Stefania Pollastro, Donato Gerin, Francesco Faretra, Rita M. De Miccolis Angelini

**Affiliations:** 1 Consiglio Nazionale delle Ricerche, Istituto per la Scienza e la Tecnologia dei Plasmi, via Amendola 122/D, 70126 Bari, Italy; 2 Academy of Sciences of the Czech Republic, Institute of Plasma Physics v.v.i., Department of Pulse Plasma Systems, Za Slovankou 1782/3, 18200 Prague, Czech Republic; 3 Department of Soil, Plant and Food Sciences, University of Bari ALDO MORO, via G. Amendola 165/A, 70126 Bari, Italy; 4 Consiglio Nazionale delle Ricerche, Istituto per la Protezione Sostenibile delle Piante, via Amendola 122/D, 70126 Bari, Italy

**Keywords:** Fungi, Applied physics, Antifungal agents, Biophysical chemistry, Optical spectroscopy, Atomic and molecular collision processes, Biological physics, Chemical physics

## Abstract

Fungal diseases seriously affect agricultural production and the food industry. Crop protection is usually achieved by synthetic fungicides, therefore more sustainable and innovative technologies are increasingly required. The atmospheric pressure low-temperature plasma is a novel suitable measure. We report on the effect of plasma treatment on phytopathogenic fungi causing quantitative and qualitative losses of products both in the field and postharvest. We focus our attention on the *in vitro* direct inhibitory effect of non-contact Surface Dielectric Barrier Discharge on conidia germination of *Botrytis cinerea*, *Monilinia fructicola*, *Aspergillus carbonarius* and *Alternaria alternata*. A few minutes of treatment was required to completely inactivate the fungi on an artificial medium. Morphological analysis of spores by Scanning Electron Microscopy suggests that the main mechanism is plasma etching due to Reactive Oxygen Species or UV radiation. Spectroscopic analysis of plasma generated in humid air gives the hint that the rotational temperature of gas should not play a relevant role being very close to room temperature. *In vivo* experiments on artificially inoculated cherry fruits demonstrated that inactivation of fungal spores by the direct inhibitory effect of plasma extend their shelf life. Pre-treatment of fruits before inoculation improve the resistance to infections maybe by activating defense responses in plant tissues.

## Introduction

Fungal diseases represent one of the main constraints to agriculture and food production, reducing product quantity and quality and causing severe economic losses worldwide. Synthetic fungicides have been largely used as powerful tools for crop protection against many fungal diseases reducing yield losses and contributing to food security. However, their excessive use implicates health and ecological risks and could lead to the selection and spread of resistant strains in fungal pathogens^1^. Therefore, the implementation of new safer and environmentally friendly solutions in crop protection are encouraged in order to minimize these risks.

Currently, there is an increasing number of undernourished people and worldwide food demand for the steady population growth^2^. In this context, the integrated use of all the available plant protection measures would play a key role in limiting yield losses caused by plant diseases both in the field as well as in postharvest. Moreover, contamination of food and feeds with toxic substances, including mycotoxins, and human pathogens like *Salmonella* spp., *Listeria monocytogenes*, *Escherichia coli*, and noroviruses represent ongoing challenges for growers and food processors^3,4^. For these reasons, new sustainable control strategies for plant and food protection are required as a valid eco-friendly possibility for improving the safety and quality of food products and reducing the impact on the environment.

Innovative technologies, based on sustainability, human safety, and long-term eco-safety, has been recently promoted and investigated. Among those, the atmospheric pressure low temperature plasma (LTP) represents a novel promising tool^5^. LTPs can be produced with different discharge configurations, such as corona, micro-hollow cathode, gliding arc, atmospheric uniform glow, dielectric barrier discharges, plasma jet and needle^6–14^.

The composition and efficacy of LTP is related to the device and system operating parameters (gas mixture, flow rate, humidity, temperature, voltage and frequency)^15–17^. Atmospheric air LTP is a mixture of electrons, ions, radicals, stable reaction products, excited atoms and molecules, and ultraviolet radiation (UV) known for their antimicrobial properties^18^. The species produced by air plasma sources are electronically and vibrationally excited molecules (O_2_ and N_2_), reactive oxygen and nitrogen species (ROS and RNS)^6^. In general ROS species trigger an oxidative stress response leading to detrimental oxidative cell damage^19,20^ and consequently to the inactivation of microorganisms^13,21–30^. Hydroxyl radicals (OH) have a direct impact on the cell membranes of microorganisms, made of a bilayer of glycerophospholipids and proteins, that are susceptible to their attacks^31,32^.

In the last two decades, extensive multidisciplinary research has been carried out proving the validity of the plasma technology application in the broad field of biology and health care^6,33–36^, dental care^37–39^, skin diseases^40^, chronic wounds^41^, cosmetics^42,43^ and antimicrobial clinical treatments against various pathogens^44^. As for plasma medicine, interest has grown in recent years on the application of plasma to agriculture, with an increasing number of uses for the treatment of crops, seeds, water and soil, and at multiple steps in the food industry. On seeds, LTP treatment reduces microbial contamination and promotes germination, rooting and growth of seedlings^45–53^. It has enhanced safety while maintaining quality properties of a wide range of perishable food products in a fast processing time^54^. LTP is a promising tool for the inactivation of microbial contaminants^55^, including foodborne pathogens^56^, and preservation of perishable food products. It has a great potential for industrial applications due to its non-thermal operation, short processing time, energy efficiency, and antimicrobial efficacy with minimal impact on food quality and the environment^57,58^.

The characteristics of target microorganisms play an important role in achieving successful decontamination with plasma treatment. Current LTP technology has significantly reduced the microbial load in *in vitro* tests on bacteria up to a 1 million factor in a few seconds. We can expect differences in sensitivity among microorganisms related to their intrinsic differences in cytology, morphology, reproductive cycles, and growth^59^. However, unlike conventional antimicrobial agents, plasma is expected to further activate the natural host defences, giving an additional chance to avoid the threat of pathogen infection^60^.

The duration of treatment and power supply for plasma generation are extremely important parameters that could affect the efficacy of the treatment^57^. Careful selection of such parameters must be performed in order to act only on microorganisms without affecting produce commercial value.

In the present paper, we report on the effects of plasma treatment on important fungal plant pathogens, *Botrytis cinerea*, *Monilinia fructicola*, *Aspergillus carbonarius* and *Alternaria alternata*, causing quantitative and qualitative losses of agricultural production both in the field and post-harvest. We focused our attention on the direct inhibitory effects of Surface Dielectric Barrier Discharge (SDBD) on spore germination of the four fungi. Different diagnostic techniques have been herein used in an attempt to understand the pathway responsible for the observed inhibitory effect. The first application of plasma treatment was also carried out on cherry fruit, in order to promote a possible application of the developed method in agricultural practice.

## Results

### Discharge system

**Figure 1:**
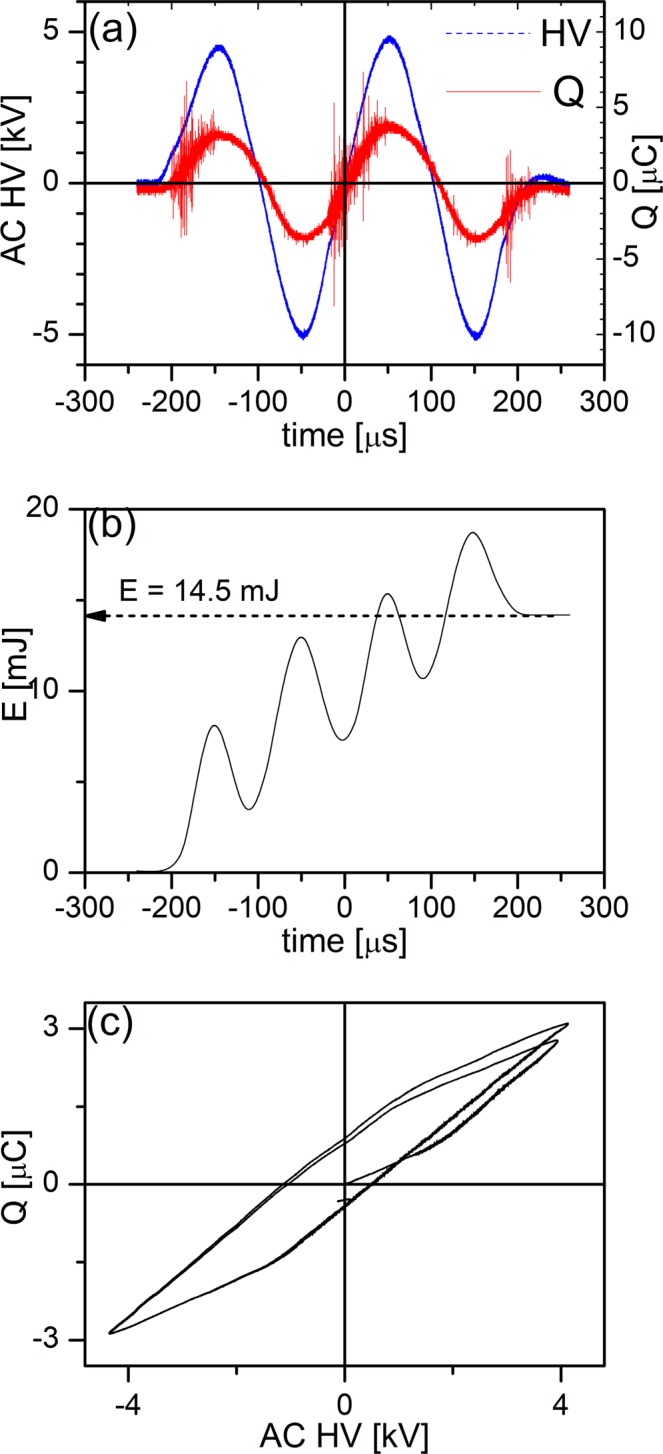
Typical applied voltage Burst and charge (**a**), energy dissipated per burst (**b**) and corresponding charge-voltage Lissajous figures (**c**).

The SDBD operated at atmospheric pressure in humid ambient air (25 °C, 40% RH). The driving AC-voltage (8.6 kV_pp_ (peak-to-peak), see Fig. 1) was a burst of two AC cycles (*f*_*AC*_ = 5 kHz) with a repetition rate of 500 Hz (duty cycle of 0.2) ensuring a homogenous distribution of microdischarges. The real discharge ‘ON’ period is actually shorter compared with the AC cycle period, we can estimate an effective duty cycle of the order of 0.1 of the nominal one^53^. This guarantees that the biological samples were not exposed to excessive noxious temperatures. The average power was 6.5 W, with an energy density of 1.5 10^−2^ Wh L^−1^. The air SDBD worked in the ozone mode, concentration typically 200–300 ppm, exceeded significantly nitrogen oxide product concentration, typically 30–40 ppm of NO_2_ and 2–3 ppm of N_2_O^13,53^.

**Figure 2:**
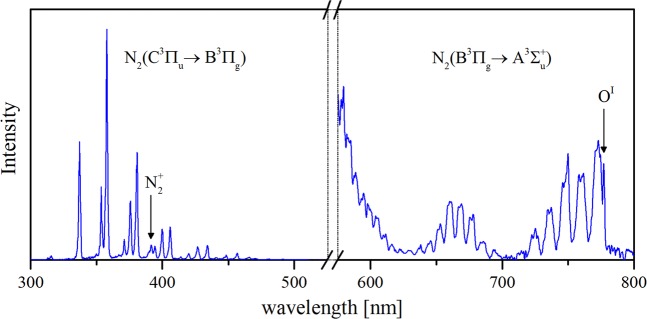
Typical averaged electron impact induced emission over multiple discharge filaments developing over two AC HV cycles. The UV and Vis-NIR parts of the spectra are not in scale.

**Figure 3:**
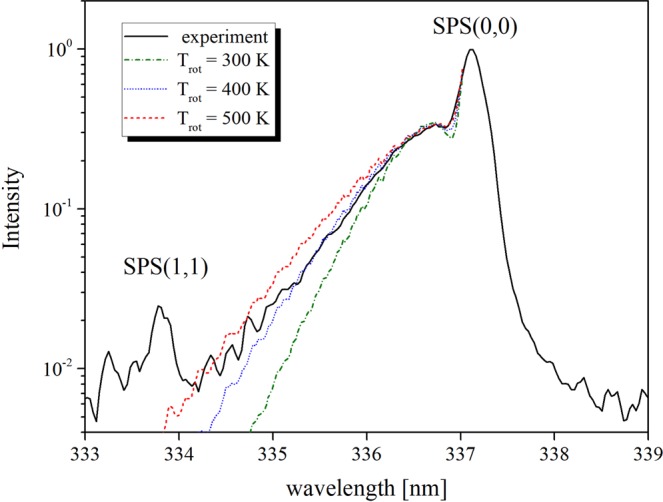
Experimental SPS(0,0) band emission (solid line) and simulated band profiles for three different temperatures.

Figure 2 shows low resolution emission spectra of filamentary SDBD driven in humid air revealing strong bands of the second positive system (SPS) (C^3^Π_u_ → B^3^Π_g_) of N_2_ and first negative system (FNS) (B^2^Σ_u_^+^ → X^2^Σ_g_^+^) of N_2_^+^ in the UV spectral range. In the Vis-NIR range, we observed characteristic sequences of bands of the first positive system (FPS) (B^3^Π_g_ → A^3^Σ_u_^+^) of N_2_. Atomic oxygen emission line was clearly observable at 777 nm, indicating the production of atomic oxygen during the plasma on phase. Partially-resolved structures of SPS displayed in Fig. 3 were analysed by means of synthetic models detailed in^61^. The SPS(0,0) band can be fitted for a given instrumental function by fixing the rotational temperature of 400 ± 25 K. We should notice that this temperature is related to the discharge on time in the surface discharge. SDBD plasma is localized just a few microns above the dielectric surface and this cannot be considered the temperature of the gas in contact with the treated substrate. Moreover, considering that the gas is flowing at 7 slm, the residence time in the discharge volume is 64 ms, therefore we can exclude a heat accumulation inside the gap. In Fig. 3 experimental SPS(0,0) band profile is shown together with two synthetic profiles (simulated for rotational temperatures of 300 and 500 K) in logarithmic scale in order to demonstrate sensitivity of the tail of the band (formed by the overlap of R_1_, R_2_ and R_3_ branches) with respect to the rotational temperature of the C^3^Π_u_ state.

**Figure 4:**
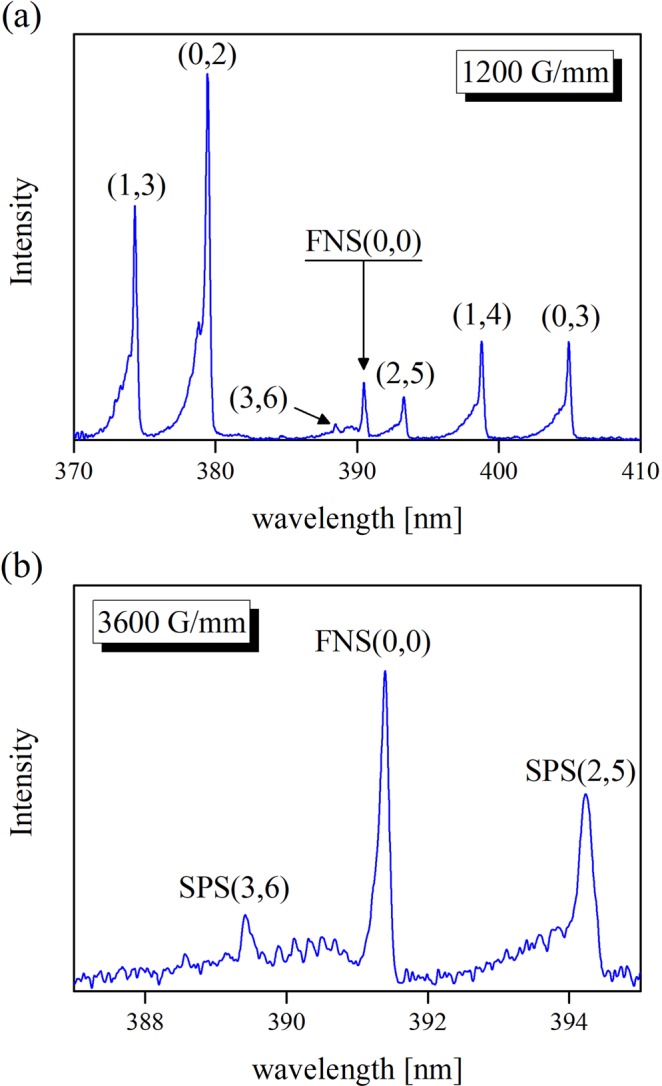
Typical electron-impact induced emission in the UV spectral range averaged over multiple discharge filaments.

Figure 4a captures the emission formed by Δv = −3 and −2 sequences of the SPS and by Δv = 0 sequence of the FNS. The ratio between FNS (0, 0) and SPS (0, 3) is equal to 0.56 ± 0.02 (Fig. 4b). Using FNS/SPS calibration curves^62^ we can estimate that the averaged reduced electric field E/N exceeds 900 Td.

**Figure 5:**
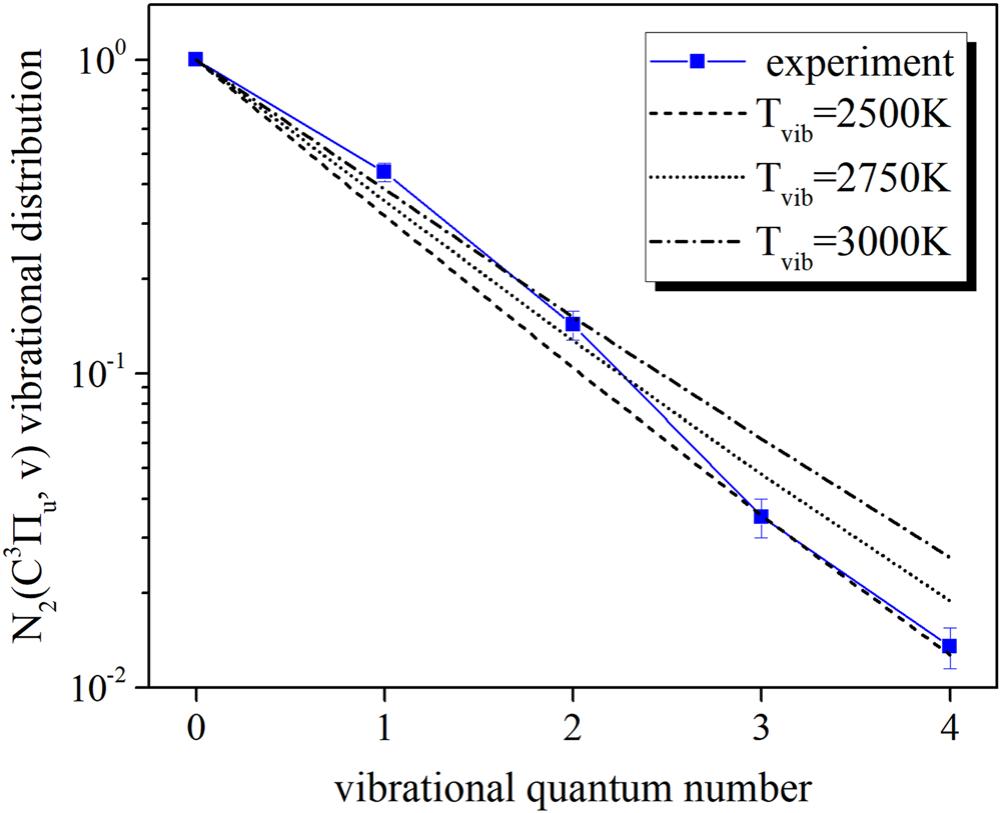
N_2_ (C^3^∏_u_) state vibrational distribution.

Figure 5 shows characteristics vibrational distributions of N_2_ (C^3^∏_u_) evaluated from time and space averaged spectra shown in Fig. 4. Furthermore, the other three vibrational distribution functions (VDFs) corresponding to the Boltzman distributions characterized by the vibrational temperature of 2500 K, 2750 K and 3000 K are shown together with the experimental data points. The VDF is characterized by the non-Boltzman vibrational distribution.

### Effects of SDBD treatments on conidial germination

**Figure 6:**
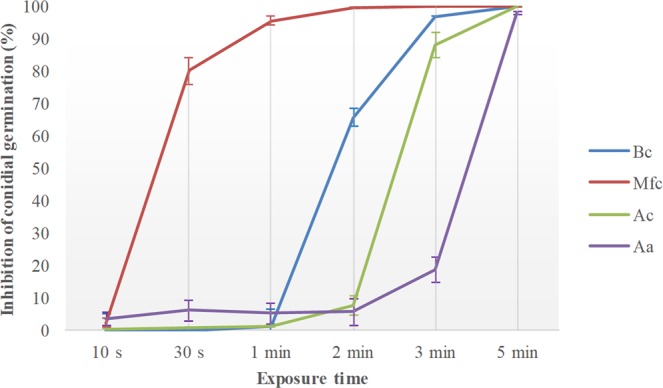
Response to SDBD exposure of *B.* *cinerea* (Bc), *M. fructicola* (Mfc), *A. carbonarius* (Ac) and *A. alternata* (Aa) in conidial germination tests.

The treatments with plasma significantly inhibited conidia germination of four different fungi exposed to SDBD (Table 1; Fig. 6).

**Table 1:** Fungal spore germination after exposure to Surface Dielectric Barrier Discharge (SDBD).

Treatment conditions	Percentages of conidial germination (± SE)							
	*Botrytis cinerea*		*Monilinia fructicola*		*Aspergillus carbonarius*		*Alternaria alternata*	
Untreated control	95.7 (±1.1)	a A	93.2 (±0.4)	a A	99.1 (±0.1)	a A	93.2 (±0.2)	a A
10 s	87.7 (±4.2)	a A	93.6 (±1.3)	a A	98.8 (±0.3)	a A	91.8 (±1.8)	a A
30 s	90.4 (±0.6)	a A	19.4 (±4.1)	b B	98.8 (±0.4)	a A	88.4 (±3.2)	a AB
1 min	89.7 (±4.9)	a A	4.3 (±1.4)	c C	98.1 (±0.2)	a A	88.0 (±3.1)	a AB
2 min	31.2 (±2.6)	b B	0.6 (±0.2)	c C	91.6 (±2.9)	a A	89.6 (±3.8)	a AB
3 min	3.0 (±0.0)	c C	0.1 (±0.1)	c C	11.9 (±3.9)	b B	77.3 (±4.3)	b B
5 min	0.0 (±0.0)	c C	0.2 (±0.1)	c C	0.0 (±0.0)	c C	2.1 (±0.4)	c C

Mean values (±Standard Error) followed by the same letter, on the column, are not statistically different at the probability levels p = 0.05 (small letters) or p = 0.01 (capital letters) with the Tukey’s HSD test.

Pre-treatment of water-agar discs before conidia inoculation did not affect their germination, although a slight reduction (4.5–11%) of germinated conidia was observed only in *B. cinerea* (data not shown). This is a clear indication that substrate chemistry modifications due to plasma interaction is not responsible for inhibition of conidia germination.

**Figure 7:**
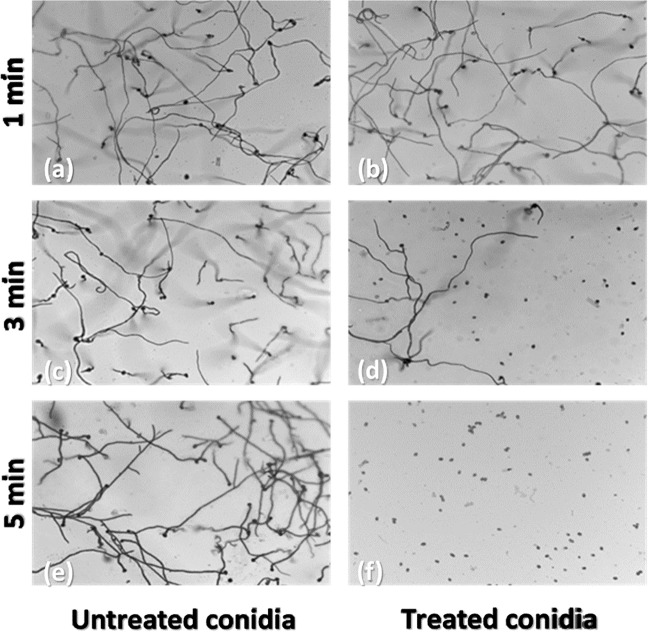
Effects of SDBD treatment on conidial germination in *B. cinerea* 18 h after inoculation. Left column (**a**,**c**,**e**), untreated conidia inoculated on treated solid media; right column (**b**,**d**,**f**), conidia treated after inoculation on untreated agar media disks.

As expected, the inhibitory effect of the treatments progressively increased with the extension of the time of exposure to SDBD. Different sensitivities in spore germination were recorded among the fungal species with *M. fructicola* found as the most sensitive being significantly inhibited (79.1%) after only 30 s of exposure. In *B. cinerea* a significant reduction (67.4%) in the percentage of germinated conidia was recorded after 2 min of treatment while a complete inhibition of the germination was reached after exposures of 5 min (Fig. 7). At least a 3 min treatment was required to obtain a significant inhibitory effect on conidia germination of *A. carbonarius* and *A. alternata*, with different efficacy for the two fungi (88.0% and 18.6%, respectively), while a complete (*A. carbonarius*; 100%) or almost complete (*A. alternaria*; 99.8%) inhibition was observed after 5 min.

### Scanning electron microscopy (SEM) observations

**Figure 8:**
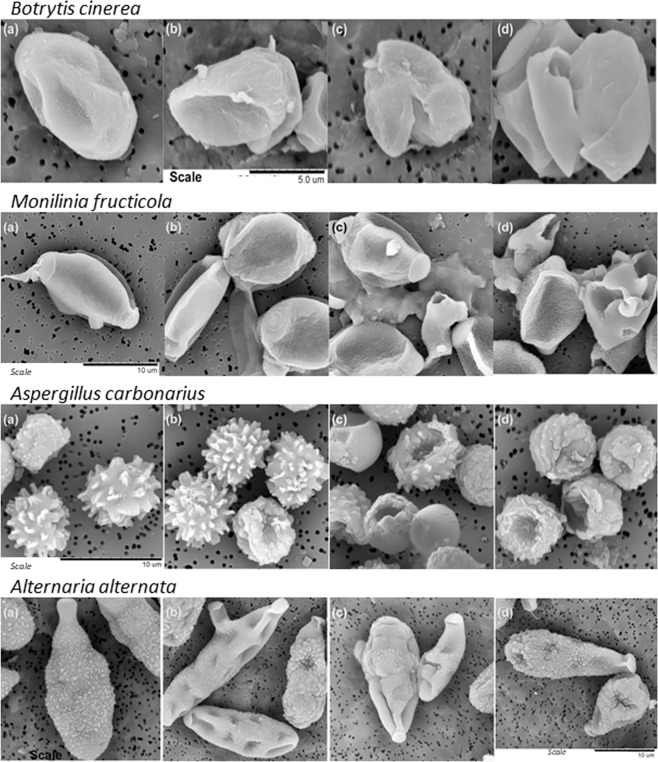
Conidia of *B. cinerea*, *M. fructicola*, *A. carbonarius*, *A. alternata*. SEM images report untreated conidia (**a**), and conidia after 1 minute (**b**), 3 minutes (**c**) and 5 minutes (**d**) of plasma treatment.

SEM analysis was performed to assess the structural damages to fungal conidia produced by plasma just after treatment (Fig. 8). *M. fructicola* conidia were confirmed as the most sensitive to plasma treatment exhibiting pronounced effects suggesting irreversible damage of the fungal structures after 1 min exposure, or even before (data not shown), corresponding to high inhibition rates recorded for this fungus in the conidial germination assay (Fig. 6). The evolution of damages on conidia surface spanned from cracks to complete removal of the outer layer of the cell wall, shrinkage, and a deeper ablation from the surface of conidia. In the case of *B. cinerea*, the early effect on the surface mostly consisted of partial ablation of the outer layers of cell wall without any crack while damages appeared irreversible after 3 min of exposure, and after 5 min most conidia were completely destroyed. In the case of *M. fructicola*, the effect was even stronger with cracks appearing after 1 min, and damages of conidia were more evident following longer treatment times. After 3 min of exposure residues of the destroyed conidia and their leaked cellular contents were visible on the polymeric membrane used as substrate material. In the case of *A. carbonarius*, the surface ornamentation of conidia was primarily affected by plasma treatment just after 1 min, and more evident after 3 min of treatment when spore walls were smoothed out by plasma ablation and cell wall integrity was obviously compromised in agreement with the results of the conidia germination assay. After 5 min of plasma exposure, conidia appeared almost completely destroyed with cracks, holes on the surface with leakages of cellular content. *A. alternata* exhibited the most resistant spores, mainly showing shrinkage and ablation processes interesting conidia wall ornamentations, while damage to the cell integrity was only achieved at the longest treatment time (5 min).

### Fluorescence-based viability assays

**Figure 9:**
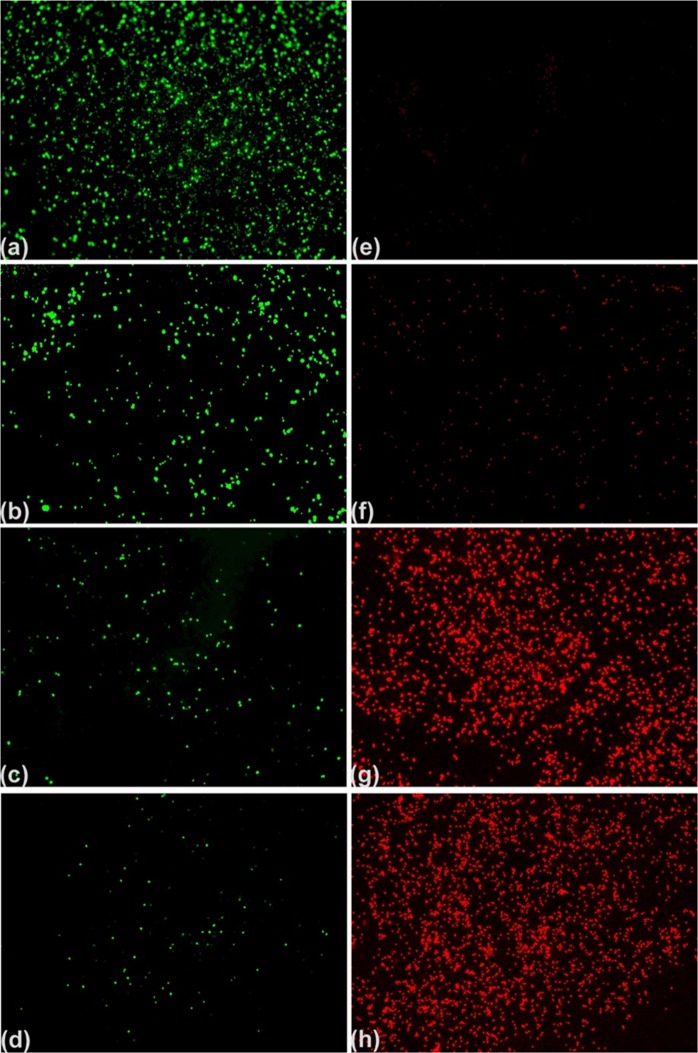
Untreated conidia of *B. cinerea* (**a**,**e**), and conidia after 1 min (**b**,**f**), 3 min (**c**,**g**) and 5 min (**d**,**h**) of exposure to SDBD stained with carboxyfluorescein diacetate (cFDA; **a**–**d**) or propidium iodide (PI; **e**–**h**).

A live/dead viability assay with carboxyfluorescein diacetate (cFDA) and propidium iodide (PI) was applied to *B. cinerea* conidia. The assay yielded results in agreement with the tests on conidial germination and SEM observations. Unexposed control conidia could be stained with cFDA, but not with PI. As expected, the number of conidia stained with PI increased with the time of exposure to the treatment while those stained with cFDA decreased, and only a few fluorescent conidia were visible after 3 min of treatment (Fig. 9). This indicates that the cell membrane integrity of the spores was affected by the treatment compromising their viability.

### Investigations on artificially inoculated cherry fruit

**Figure 10:**
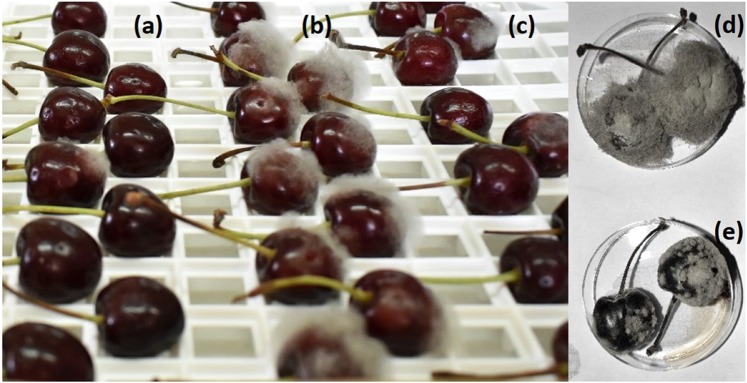
Cherry fruits artificially inoculated with *B. cinerea* 6 days after inoculation (5 min-SDBD treatment (**a**), untreated control (**b**), 1 min-SDBD treatment (**c**)); infections of *B. cinerea* (**d**) and *M. fructicola* (**e**) on untreated control fruits 10 days after inoculation.

The efficacy of SDBD against *B. cinerea* and *M. fructicola* infections on fresh fruits was assessed in *in vitro* assay on cherry fruit following artificial inoculations (Fig. 10).

**Figure 11:**
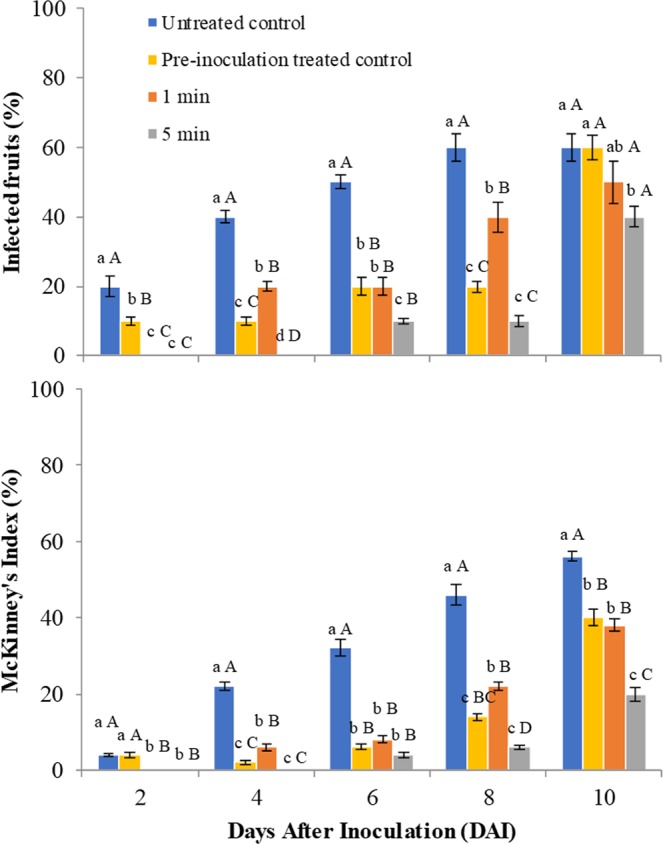
Effect of SDBD treatments on cherry fruit cv. Sweet heart artificially inoculated with *B.* *cinerea* (1 × 10^5^ conidia mL^−1^) during 2017. Bars represent standard error; treatments with the same letter are not statistically different at the probability levels p = 0.05 (small letters) or p = 0.01 (capital letters) with the Tukey’s HSD test.

During 2017, a preliminary evaluation was carried out on both pre- and post-inoculation treatments, using *B. cinerea* as inoculum and symptom assessments at 5 different days after inoculation (DAI) (Fig. 11). Until 8 DAI, all treatments significantly (*p* = 0.01) reduced both disease prevalence (P) and McKinney’s Index (MKI) as compared to the untreated control. In detail, at 2 DAI symptoms were recorded on the untreated control (P = 20%; MKI = 4%) and on fruit treated with 3-min SDBD exposure before pathogen inoculation (P = 10%; IMK = 1%). The pre-inoculation treatment showed an efficacy (measured as Abbott’s Index, AI) up to 75% (P) and 91% (MKI) at 4 DAI and was also able to significantly (*p* = 0.01) reduce disease incidence (AI = 29%) but not prevalence at 10 DAI. On fruits exposed after inoculations to 1 and 5 min of treatment, symptoms appeared from 4 (P = 20%; IMK = 6%) and 6 (P = 10%; IMK = 4%) DAI, respectively. Higher efficacy was obtained following 5-min exposure with AI ≥ 80% until 8 DAI and the treatment significantly reduced disease prevalence (*p* = 0.05; AI = 33%) and incidence (*p* = 0.01; AI = 64%) even at 10 DAI (Fig. 11).

**Figure 12:**
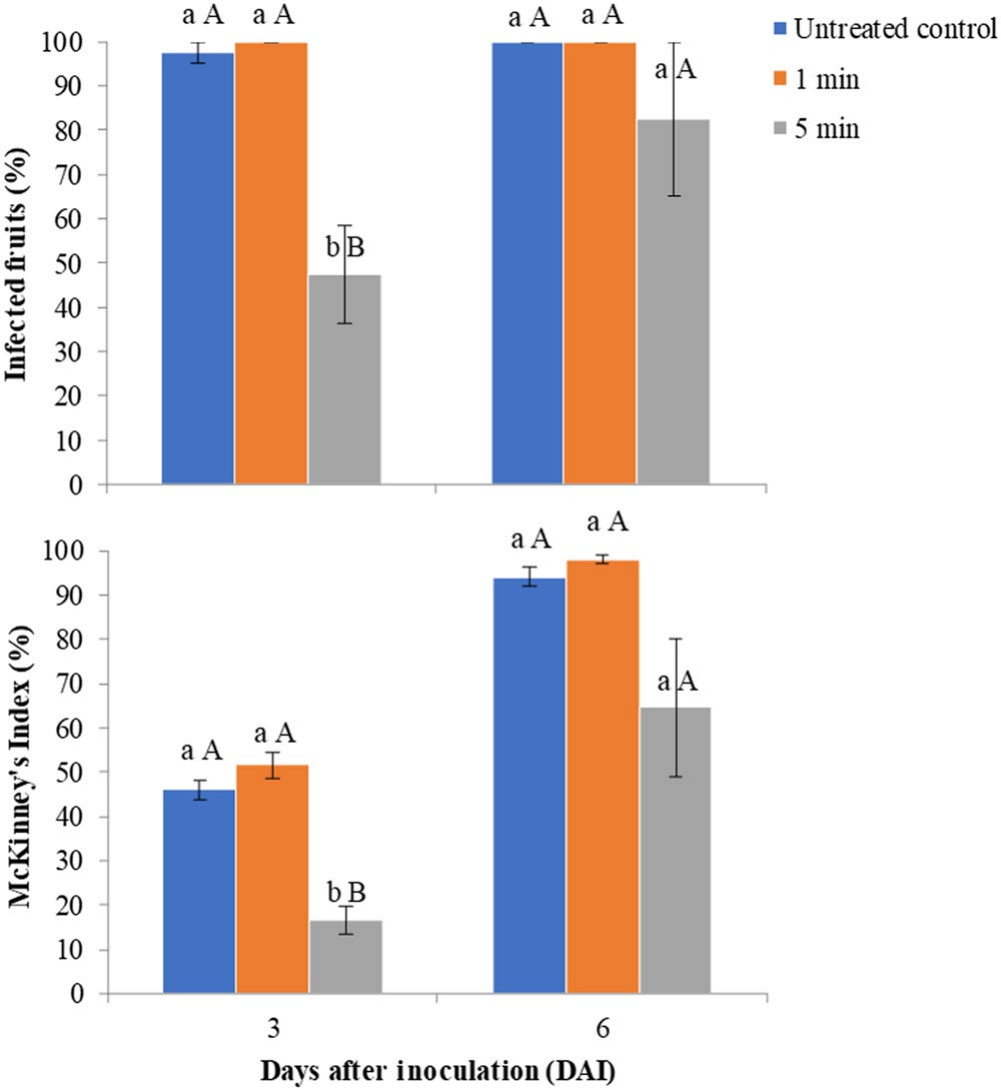
Effect of SDBD treatments on cherry fruits cv. Giorgia artificially inoculated with *B. cinerea* (5 × 10^5^ conidia mL^1^) during 2018. Bars represent standard error; treatments with the same letter are not statistically different at the probability levels p = 0.05 (small letters) or p = 0.01 (capital letters) with the Tukey’s HSD test.

During 2018, artificial inoculations with *B. cinerea* were conducted using higher levels of inoculum than in the previous experiment. At 3 DAI, the incidence of grey mould on inoculated fruits was high on both the untreated control (P = 97.5%; IMK = 46.0%) and after 1 min of exposure to SDBD (P = 100%; IMK = 51.5%), while a significant (p ≤ 0.01) reduction of infections was recorded on 5 min-treated fruit (P = 47.5%; IMK = 16.5%). Disease incidence increased at 6 DAI when symptoms were recorded on 100% of untreated or 1 min-treated fruit with 94–98% of MKI, while 82.5% of 5 min-treated fruit were infected and MKI was 64.5% (Fig. 12).

**Figure 13:**
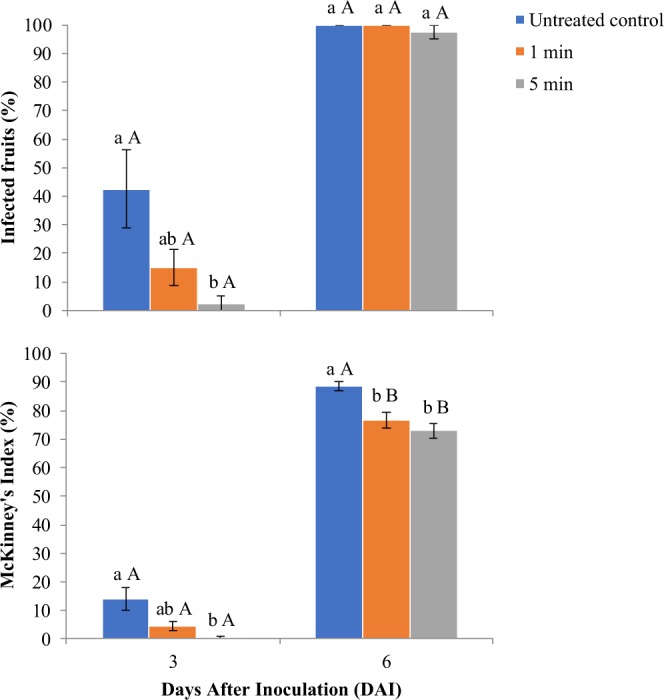
Effect of SDBD treatments on cherry fruits cv. Giorgia artificially inoculated with *M. fructicola* (5 × 10^5^ conidia mL^−1^) during 2018. Bars represent standard error; treatments with the same letter are not statistically different at the probability levels p = 0.05 (small letters) or p = 0.01 (capital letters) with the Tukey’s HSD test.

On fruits artificially inoculated with *M. fructicola*, symptoms of brown rot were recorded at 3 DAI with low incidence on 1 min (P = 15%; IMK = 4.5%) and 5 min-treated fruit (P = 2.5%; IMK = 0.5%), statistically different (p ≤ 0.05) from the untreated control (P = 42.5%; IMK = 14.0%). At 6 DAI, disease incidence always reached P values close to 100%, although IMK values on treated fruits (73.0–76.5%) were statistically (p ≤ 0.01) lower than the value of untreated control (88.5%) (Fig. 13).

## Discussion

Low temperature plasma (LTP) is a fast, economic and eco-friendly^63^ technology that can be applied to products improving their commercial quality. It creates a lethal environment for pathogenic bacteria or other types of unwanted microorganisms like molds^30,53,64^ without any residues or harmful by-products for the environment or the commodity being treated.

Mechanisms of action of LTP are completely different than those of classical decontamination techniques. The antimicrobial activity can be achieved either by UV irradiation leading to the erosion of microorganisms through photon-induced desorption that results from UV photons breaking chemical bonds in cell molecules with the formation of volatile compounds or by etching due to highly energetic ions and reactive species through bombarding and reacting with the surface of the treated materials^65^. Once the outer layers of the cell are damaged and then cellular integrity compromised, the UV photons can directly hit genetic material leading to DNA damage with the highest killing rate^66^. Direct DNA damage by direct UV irradiation of microbial cells or spores cannot lead to sterilization due to the minimal depth of penetration of UV photons into cells stacked or covered with various debris. In atmospheric pressure air plasmas, reactive species such as O, OH, and NO_2_ are believed to play the most crucial role in decontamination from microorganisms. Heat and UV radiation may play secondary roles, and we expect their effects to be either minimal or indirect.

In the present work, we observed different survival rates of spores from different fungal species exposed to SDBD plasma requiring different treatment times to reach significant inhibition of spore germination, reduction of viability and morphological alterations of cell surface up to spore destruction.

The reactive species generated by air plasmas are expected to greatly compromise cell integrity^67^. ROS and RNS have a direct impact on the cells of microorganisms, and especially on the outermost layer, the polysaccharide-rich wall which envelopes microbial cells. Plasma etching causes the breakdown of bonds, particularly for hydrocarbon compounds. Atomic oxygen and ozone easily react with these open bonds, which facilitates a faster etching of molecules^68–70^. This, in turn, leads to morphological changes of the cell, ranging from reduction in size to the appearance of deep channels, up to complete cellular destruction. The components of the innermost part of the cell envelope, the membrane, are well known to be the targets of oxidation chemistry^71,72^ with lipids (mostly unsaturated fatty acids) and proteins highly susceptible to attacks by hydroxyl radical (OH)^31^. This can potentially lead to alterations in overall membrane properties, including stability and permeability.

Many experiments found that low temperature plasmas can inhibit the growth of clinical^44,73^, food contaminant^35,74,75^ and phytopathogenic fungi^76^. Under comparable conditions, however, the fungicidal effect of plasma is weaker than the bactericidal effect due to the differences in structure and composition of prokaryotic and eukaryotic cells. The fungal cell wall is basically made up of chitin, α and β-linked glucans, glycoproteins and pigments^77,78^. Pigments like melanins may accumulate in fungal structure to protect them from damaging agents such as UV, ionizing, and gamma irradiation, dehydration, extreme temperatures, the action of hydrolytic enzymes, antifungal drugs and free oxygen radicals^79^.

Melanin may be contained at the cell surface or released to the extracellular space, but its exact location and abundance vary between species. In *Aspergillus* spp. belonging to the group Nigri, like *A. carbonarius*, melanins of the dihydronaphthalene (DHN) type are responsible for the dark-brown pigmentation of conidia^80^ and proved to be involved in their resistance to environmental stress and as virulence factor^81^. Melanin increases conidia resistance to reactive oxygen species in *Aspergillus fumigatus*^82^. In the multicellular conidia of *Alternaria* spp., melanin is confined to the outer region of the primary cell walls and the septa while secondary cell walls are unmelanized^83^. DHN melanin also accumulates in the conidiophores, conidia and sclerotia of *B. cinerea*, giving them their characteristic color as well as in *M. fructicola* which produces melanized conidia, and fruit mummy consisting of stroma with a highly melanized layer of surface hyphae^84,85^.

In our experiments, the dark-colored conidia of *A. carbonarius* and *A. alternaria* showed higher resistance to the treatment in comparison with lighter pigmented and thinner cell walled *B. cinerea* and *M. fructicola* conidia. We can assume that UV photons in the plasma generated at atmospheric pressure should be not enough to have a significant influence on direct inactivation of the fungi because of the protective action of melanin and similar compound embedded in the cell wall. Differences in efficacy of surface etching are more likely related to the cell wall structure and thickness affecting mechanical and chemical strength, as well as to the stacking of spores on their surfaces and the number of cells per spore. Multicellular spores may still have the possibility to germinate if not all the cells in the spore are killed by the treatment. This reduces the overall inhibitory effect on multicellular spores of some fungi, including *Alternaria* spp.

Our results by SEM analysis showed major structural damages to all the observed fungal conidia after plasma treatment, with etching and perforation of cell walls. Evident disruption of cell walls was noted in the most sensitive species *B. cinerea* and *M. fructicola* and at a lower degree in *A. carbonarius*, thus promoting cellular death. In the most resistant *A. alternata*, we observed conidia shrinkage, surface changes and cell envelope invaginations. Lysis followed by leakage of cellular content was the main destructive process observed. This is consistent with the decrease in viability of conidia and the increase in their membrane permeability showed by the fluorescence-based assays indicating leakage of cell content and subsequent cell death. Therefore, SEM analysis demonstrated modifications of the external shape of conidia, which could be attributed to the decomposition of organic components by etching and photo-desorption associated with chemical bond breakage, and consequent formation of volatile compounds^65^.

Until now, few studies have investigated LTP to control postharvest fungal diseases^5^. Indirect plasma treatments have been successfully applied to inactivate *Aspergillus ochraceus*, *Penicillium expansum* and *P. digitatum*^86,87^ and other fungi and yeast contaminating fresh produce like fruits and vegetables^88^.

In addition to direct antimicrobial activity on fresh produce, LTP may also act indirectly by inducing plant defense responses^47^. The activated chemical species produced by LTP may contribute to both the suggested modes of action by acting rapidly against contaminant microorganisms and as a defense inducer in plants. ROS and RNS have a significant impact on plant cells causing many physiological, biochemical, molecular, and hormonal changes^89^. Treatments with plasma-activated water induce plant growth as well as endogenous production of ROS and RON, defense hormone metabolism, and up regulation of pathogenesis-related genes in tomato seedlings^90^, thus enhancing resistance against bacterial leaf spot^91^. Our results suggest modifications in fruit tissues which can suggest metabolic changes affecting infection establishment. A slight colorimetric change was observed on treated fruits suggesting an increase of contents in polyphenolic compounds after plasma treatment (data not shown). Moreover, the activation of defense responses was indicated by the significant reduction of disease incidence recorded up to 8 DAI on fruits treated before pathogen inoculation.

The consistence and morphology of fruit surface is a challenging point in SDBD plasma decontamination process that requires optimization of the reactor and electrode configurations in order to improve the treatment effectiveness. The quite complex structure and consistence of some fruit tissues, like those of strawberry or blueberry, could hinder full exposure of microorganism cells to plasma effects and create physical barriers that significantly reduces the efficacy of LTP inactivation sometimes causing lesions on the fruit surface that may function as primary infection sites for wound pathogens. On the contrary, smooth and impervious surfaces such as those of cherry fruits or grape, blueberry or currant berries could facilitate deactivation of contaminant spores^56,92^. The configuration and discharge parameters used in our experiments, even if not completely optimized, allowed to extend the shelf life of cherry fruits of two different cultivars artificially inoculated with two major pathogens (*B. cinerea* and *M. fructicola*) affecting fruit quality during postharvest, processing, and storage. These results are comparable to those reported for biocontrol agents or resistance inducers used for the control of postharvest decay of fresh products^93^. They show a promise for practical application of novel nonthermal plasma technology for the treatment of fresh produce that would allow longer shelf life, less spoilage, and safer products.

## Conclusions

In summary, low-temperature air atmospheric pressure plasma represents a quite interesting and innovative control method, alternative to traditional chemicals against plant pathogens, including fungi, particularly those that cause postharvest diseases as well as seed-borne pathogens and mycotoxigenic fungi. We demonstrated that remote plasma treatment by SDBD is effective against conidia of fungi responsible for major economic losses in the agriculture industry, reducing to undetectable levels their viability/germinability on the surface of artificial media. Cell wall disruption and leakage of cell contents were caused by plasma treatments. This demonstrates that treatment can act directly on fungal spores, likely due to the combined effect of active chemical species (*i.e*., ROS and RNS) and UV lights.

Even if the SDBD device has not been optimized for the treatment of fresh fruit we obtained significant reductions in grey mould and brown rot symptoms on artificially inoculated cherries leading to an increase of their shelf life. Moreover, plasma treated fruits increased their early resistance to infections as a possible consequence of activation of defence responses in the exposed fruit tissues. Further studies will address the feasibility for scale-up of this technology to prototypal scale and commercial scale.

To improve the decontaminant effect on fruit the discharge electrode must be designed to guarantee a homogeneous plasma dose distribution on the surface reaching all the inoculum responsible for infection start up.

## Methods

### Discharge system

**Figure 14:**
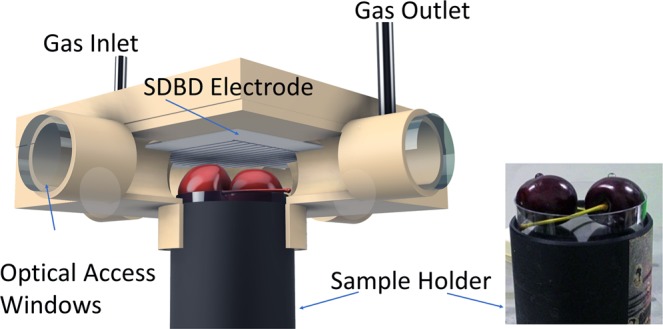
Experimental SDBD setup. The sample holder can accommodate petri dishes containing either cherry fruit or substrates inoculated with conidia. On the right the real sample holder with two cherry showing the inoculation spots.Schematic of the reactor created with Autodesk Fusion 360, ver. 2.0.7046 (https://www.autodesk.com/products/fusion-360/students-teachers-educators).

The discharge system includes the modified SDBD reactor used to study the production of active species by SDBDs^11^, gas feeding unit, discharge energization system, electrical and optical diagnostics and sample holder suitable for inserting biological samples at a selected distance from the discharge surface^53^. The SDBD reactor (Fig. 14, left) consists of a planar SDBD electrode system placed in a PVC chamber equipped with gas feed input/output ports and a high voltage (HV) interface. This reactor chamber allows the insertion of a sample holder (Fig. 14, right) next to the SDBD surface. The grounded and powered electrodes were deposited on both sides of a thin alumina substrate.

The nickel-based electrode exposed to the discharge consists of 17 parallel strips (1 mm wide, 75 mm long and separated by 3 mm)^11,94^. A silver-based induction electrode (74 mm × 74 mm) covers the opposite surface of the alumina plate. The SDBD was powered by an AC power supply composed of the TG1010A Function Generator (TTi), Powertron Model 1000 A RF Amplifier and a high-voltage step-up transformer. Applied AC HV waveform (5 kHz) was amplitude-modulated by a square-wave modulation waveform (f_M_ = 500 Hz) producing 5 kHz sine-wave T_ON_ and T_OFF_ periods with a fixed duty cycle D = T_ON_/(T_ON_ + T_OFF_) = 20% and a precise number of sine-wave bursts was selected through an external trigger realized by means of a TG 5011 Function Generator (TTi). We used a LeCroy LC534A digitizing oscilloscope (bandwidth 1 GHz, up to 2 GS/s) to record the voltage–charge, voltage–current characteristics of the discharge. We used a Tektronix P6013A high voltage probe (1,000:1@1 MW, bandwidth 25 MHz) to monitor voltage waveforms. We used a voltage probe (10:1@10 MW, bandwidth 1 GHz) to measure the potential drop on a current sensing shunt resistor (R = 10 Ω) or on a transferred charge measuring capacitor (C = 0.5 μF, both inserted alternately between the induction electrode and ground. The reactor was fed with humid ambient air (RH 40%, T = 25 °C) at a fixed flow rate of 7 slm.

### Emission spectroscopy

UV grade quartz frontal windows allow direct visual control of the discharge area and optical emission diagnostics. Andor iStar intensified charge-coupled device (ICCD) DH740i-18U-03 camera was used to register the plasma-induced emission (PIE) collected in this work from the whole SDBD surface. The ICCD was used to register the time-averaged emission spectra through the iHR-320 spectrometer (Jobin-Yvon) equipped with the 150, 1200, 3600 G/mm dispersion gratings. The intensities of the emission spectra acquired by the ICCD detector were corrected for a given detector sensitivity and optical path by means of DH-2000 deuterium-halogen light source (Ocean Optics)^53^.

### Fungal isolates and conidial germination tests

The inhibitory effect of SDBD plasma treatment against *B. cinerea*, *M. fructicola*, *A. carbonarius* and *A. alternata* was evaluated through conidial germination assays^95^ at six different exposure time. Single pure isolates of the four species were obtained from the collection of the Plant Pathology section of the Department of Soil, Plant and Food Sciences, University of Bari (Italy). The cultures, stored at −80 °C, were revitalized on potato dextrose agar (PDA: infusion from 200 g peeled and sliced potatoes kept at 60 °C for 1 h, 20 g dextrose, adjusted at pH 6.5, 20 g agar Oxoid No. 3; per liter). Conidia were collected by scraping the surface of seven-day-old colonies grown on PDA at 21 ± 1 °C, exposed for 12 h per day to a combination of two daylight (Osram, L36W/640) and two near-UV (Osram, L36/73) lamps, then suspended with a sterilized loop in sterile distilled water and filtered through glass wood to remove mycelial fragments and conidiophores. Aliquots (10 μL) of conidial suspension (0.5–1 × 10^5^ conidia mL^−1^) were spotted on discs (6 mm diameter) of water agar (WA: 20 g L^−1^ of agar Oxoid No. 3), placed on sterile microscope slides and submitted to the plasma treatment. The overall treatment times used were 10 s, 30 s, 1 min, 2 min, 3 min and 5 min. Treated WA disks inoculated with conidia unexposed to SDBD were used as an additional untreated control. The discs were then incubated in a moist chamber at 21 ± 1 °C in the dark, and after 18 h, conidia were fixed with lactophenol cotton blue. Random samples of 100 conidia on each of three replicated spots per condition were observed at ×200 magnification, and germinated conidia were counted. The inhibition of conidial germination was calculated considering the frequency of germinated conidia on untreated control medium.

### Morphological observations on plasma treated spores by scanning electron microscopy (SEM)

The effect of plasma on conidial morphology was analyzed collecting microscopic observations by a Hitachi TM3000 SEM (at 10^−4^ Pa pressure and 15 kV acceleration voltage) as described in a previous study^53^ in either Observation or Analysis mode. For best image results, samples were previously gold-palladium sputter coated for 120 s (at 10^−1^ Pa pressure, 10 kV acceleration voltage at 15 mA) by an Edwards Sputter Coater. Polymeric membranes (Nuclepore Polycarbonate, Whatman, Maidstone, UK) with the diameter and pore size of 13 mm and 0.4 µm, respectively, were used as a substrate material for fungal spores by loading conidial suspension (1 × 10^6^ spores mL^−1^) from *B. cinerea*, *M. fructicola*, *A. carbonarius* and *A. alternata*.

### Fluorescence-based viability assays

The effect on viability and cell membrane integrity was carried out by using the fluorescent probes carboxyfluorescein diacetate (cFDA) and propidium iodide (PI). cFDA acts as a substrate for enzyme activity, that can cross cell membranes. Once inside, it is cleaved by non-specific esterases to release the green-fluorescent carboxyfluorescein, which the cell retains inside. Thus, cell viability can be correlated with its ability to accumulate carboxyfluorescein^96^. In contrary, the red-fluorescent nucleic acid probe PI can only enter cells that have damaged membranes and is generally excluded from viable cells^97^. A stock solution of cFDA (Sigma‐Aldrich, Milan, Italy) was prepared in acetone (4.6 mg mL^−1^) and stored at −20 ± 2 °C in the dark. A stock solution of PI (Sigma‐Aldrich; 0.67 mg mL^−1^) was prepared in distilled water and stored at 4 ± 1 °C in the dark. Suspensions of *B. cinerea* conidia were loaded on polymeric membranes as previously described and exposed to plasma. Treated and untreated samples were then incubated with cFDA and PI at a final concentration of 10 µM for 15 min at 25 °C, and the staining of conidia determined visually by microscopy observation. The fluorescence microscope DM5500 (Leica, Wetzlar, Germany) was equipped with an external light source Leica EL6000 and the following filter systems: L5 for blue excitation (excitation filter BP 480/40, dichromatic mirror 505, suppression filter 527/30) and N2.1 for green excitation (excitation filter BP 515–560, dichromatic mirror 580, suppression filter LP590).

### Investigations on artificially inoculated cherry fruit

The effectiveness of plasma treatments was evaluated in two different trials on freshly harvested cherry fruits. During 2017, unblemished cv. Sweet heart cherry fruits were wounded with a sterile needle (approximately 4 mm in depth), placed in sterile Petri dishes (55 mm diameter; two replicated fruits per dish) and inoculated at the equatorial region with 10 µL of conidial suspensions (1 × 10^5^ spores mL^−1^) of *B. cinerea*. During 2018, selected cv. Giorgia cherry fruits were wounded and each was artificially inoculated at five equidistant points along the equatorial area with 10 µL of conidial suspensions (5 × 10^5^ spores mL^−1^) of *B. cinerea* and *M. fructicola*. After inoculations, fruit surfaces were exposed to SDBD at two different treatment times: 1 or 5 min. Each treatment was conducted on four replicated groups of ten fruits while untreated inoculated and mock fruits inoculated with sterile water served as control checks. Fruits were put inside humid chambers, incubated at 21 ± 1 °C and grey mould or brown rot symptoms were assessed at the following days after inoculation (DAI): (i) 2, 4, 6, 8 and 10 (first trial, 2017); (ii) 3 and 6 (second trial, 2018). Evaluations were carried out by using the following empirical scale: 0 = healthy fruit; 1 = 1–5% of infected fruit surface; 2 = up to 25%; 3 = 26–50%; 4 = 51–75%; 5 = 76–100%. The usage of the empirical scale allowed the calculation of the following parameters: 1) Prevalence (P; percentage of infected fruits); (2) McKinney’s Index $(IMK=\frac{\sum (f\cdot v)}{N\cdot X}\cdot 100)$, where: *f* = frequency of cases falling in each class; *v* = class value; *N* = total number of observed cases. Abbott’s Index (AI) was calculated for data of P and MKI of each assessment with the formula: AI = [(Xuntreated − Xtreated)/Xuntreated] × 100.

### Statistical analysis

Data were analyzed by ANOVA using the statistical program CoStat software version 6.45 and Tukey’s HSD (Honestly Significant Difference) procedure to compare all pairs of means at 0.05 and 0.01 confidence interval (p).
